# Sympathetic Response of Children With Autism Spectrum Disorder During Dental Treatment Performed in a Sensory-Adapted Dental Environment

**DOI:** 10.7759/cureus.66685

**Published:** 2024-08-12

**Authors:** Akansha Kaintura, Kavitha Ramar, U. Ganapathy Sankar

**Affiliations:** 1 Pediatrics and Preventive Dentistry, Sri Ramaswamy Memorial Kattankulathur Dental College, Chennai, IND; 2 Pedodontics and Preventive Dentistry, Sri Ramaswamy Memorial Kattankulathur Dental College, Chennai, IND; 3 Occupational Therapy, Sri Ramaswamy Memorial Institute of Science and Technology, Chennai, IND

**Keywords:** occupational therapy, electrodermal activity, sensory adapted dental environment, sensory evaluation, sensory in autism and anxiety, autism spectrum disorder and emotion

## Abstract

Autism spectrum disorder (ASD) is a neurodevelopmental condition characterized by difficulties in social interaction, communication, and sensory processing. These challenges often make dental visits overwhelming and distressing for children with ASD. This study explores the use of electrodermal activity (EDA) to measure physiological stress responses and evaluates strategies to enhance cooperation during dental treatments in a sensory-adapted dental environment.

We conducted a case series involving three children with ASD who required dental treatment. Each child's physiological responses to dental stimuli were monitored using EDA, which measures changes in skin conductance levels and skin conductance responses. Interventions included the use of dim lighting, the avoidance of loud noises, the application of firm pressure, the provision of sensory toys, social stories before appointments, and desensitization and video modeling techniques.

All three patients exhibited phasic variations in EDA levels in response to stressful stimuli and tonic changes with calming stimuli. Case 1 responded to bright lights and unfamiliar settings with increased phasic activity, while calming stimuli like firm pressure resulted in tonic changes. Case 2 showed similar phasic responses to a weighted lap pad and tonic changes with music. Case 3 reacted to confined spaces and sudden light and touch with phasic variations and both a massager and music-induced tonic changes. Interventions were tailored to each patient's specific stressors, resulting in improved cooperation and reduced stress levels.

The study demonstrates the effectiveness of EDA as a tool for monitoring stress responses in children with ASD during dental treatments. Tailoring interventions to individual sensory needs can significantly enhance patient cooperation and comfort. These findings highlight the importance of adapting dental environments and protocols to accommodate the unique needs of children with ASD, with collaborative efforts from occupational therapists and dentists.

## Introduction

Neurodevelopmental disorders, such as autism spectrum disorder (ASD), are characterized by repetitive behaviors, restricted interests, and challenges in social interactions. ASD is a neurobiological condition influenced by both environmental and genetic factors that affect brain development [1]. Children with ASD often experience difficulties receiving dental treatment because of various sensory sensitivities and behavioral challenges [2]. These challenges can make dental visits overpowering and anxiety-inducing for both the child and the dental team [3]. To provide effective dental treatment for children with autism, a sensory-adapted dental environment can be created. This environment takes into consideration the sensory needs of the child and provides a more comfortable and accommodating setting for dental procedures. The introduction of deep pressure, rhythmic music, and reduced bright light may reduce unfavorable patient reactions and increase participation in dental treatments [3]. Cermak et al. concluded that using a sensory-adapted dental environment demonstrates utility and positive treatment effects. This approach has the potential to enhance dental care not only for children with ASD but also for those with other disabilities and typically developing children with dental anxiety and/or sensory processing difficulties. Further research is needed to identify factors that may influence treatment efficacy [3].

Furthermore, measuring a child’s electrodermal activity (EDA) response, which can be described as the electrical conductance of the skin, which is influenced by the activity of the eccrine sweat glands, may be done non-invasively using the EDA response. Since sympathetic cholinergic fibers innervate these glands primarily, skin conductance is hypothesized to offer a relatively "undiluted" measure of sympathetic activity [4]. An involuntary function that is measured by changes in the electrical properties of the skin due to underlying medical conditions or stressors can offer important insights into the child’s physiological responses during dental treatment [4]. By evaluating physiological stress and anxiety, measured by EDA, caused by various stress stimuli in patients. We can help patients cooperate better during dental treatment.

Phasic change and tonic change are the two categories into which an EDA response falls. Skin conductance responses (SCRs), which are fast phasic components resulting from sympathetic neural activity, and background tonic (skin conductance level (SCL)) components make up EDA. SCL stands for the tonic component, which is related to the background features and slower-acting elements of the signal, such as the general level and gradual variations over time. Variations in SCL correspond to overall autonomic arousal [5]. The smooth, gradual changes in the EDA response signal that occur in the absence of stressful stimuli are referred to as tonic changes or SCL [5]. The signal's faster-changing components are referred to as the phasic component, which is symbolized by SCR. According to recent data, SCL and SCR may depend on distinct brain pathways and are both significant. Due to its independence from parasympathetic activity, EDA is perhaps the most effective indicator of changes in sympathetic arousal associated with emotional and cognitive states. EDA is frequently utilized as a sensitive indicator of autonomic emotional and cognitive processing as well as sympathetic activity. It has been intimately associated with these activities [5].

To enhance the prospects for children with ASD in the dental clinic, interdisciplinary cooperation with other domains (occupational therapy, psychology, computer science behavioral therapy, special education, and informatics) provides diverse viewpoints on approaches and interventions [6]. These tactics include educating individuals about sensory sensitivity, creating a sensory-friendly environment, assisting in the development and use of visual aids and social narratives, and utilizing virtual and video technologies. All these tactics have at least some early evidence to support their application for children with ASD [6].

This case series aimed to analyze the sympathetic responses of children with ASD during dental treatment performed in a sensory-adapted dental environment.

## Case presentation

Case 1

Patient Description

**Figure 1:**
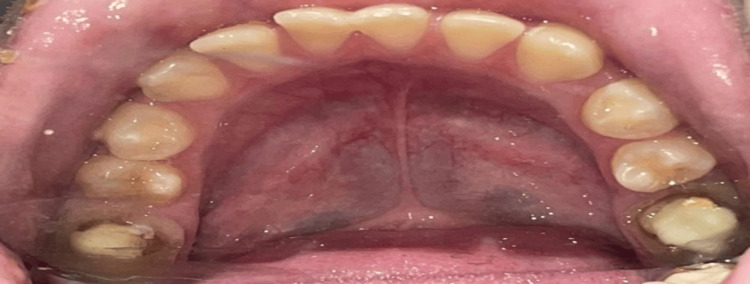
Preoperative clinical photograph A preoperative clinical photograph showing the oral cavity of a 12-year-old girl with ASD requiring a dental prosthesis for her left and right first mandibular teeth, which had undergone root canal therapy. ASD: autism spectrum disorder

A 12-year-old girl with a moderate degree of ASD came to the Department of Pediatric and Preventive Dentistry in need of a dental prosthesis for her left and right first mandibular teeth, which had undergone root canal therapy. Her mother expressed concerns regarding her oral hygiene habits and sensitivity to sensory stimuli during previous dental visits. Figure 1 shows a preoperative clinical photograph of the oral cavity. Her mother also reported that dental treatment had been performed under general anesthesia six months prior.

She had a history of sensory sensitivities, particularly to bright lights, loud noises, and unfamiliar environments. She had difficulty with transitions and exhibited repetitive behaviors when anxious. Her previous dental visits were challenging because of her sensory issues, resulting in incomplete examinations and prophylaxis when done chairside.

During the initial examination, she exhibited signs of dental plaque accumulation, particularly in the posterior regions and root canal-treated teeth in the mandibular left and right first molars. Her oral hygiene appeared suboptimal, likely due to difficulty with toothbrushing and oral care routines at home. She showed hesitation to open her mouth fully and exhibited signs of anxiety, such as rocking back and forth, avoiding eye contact, and showing distress by screaming.

Treatment Plan

Pertaining to the patient’s history and specific needs, a comprehensive treatment plan was developed in collaboration with her parents and behavioral therapist. The plan focused on creating a sensory-friendly environment and implementing behavioral strategies to facilitate successful dental treatment. Prior to the appointment, her parents were provided with visual aids and social stories explaining the dental visit process. The dental team communicated with her using clear, concise language and provided reassurance throughout the appointment.

**Figure 2:**
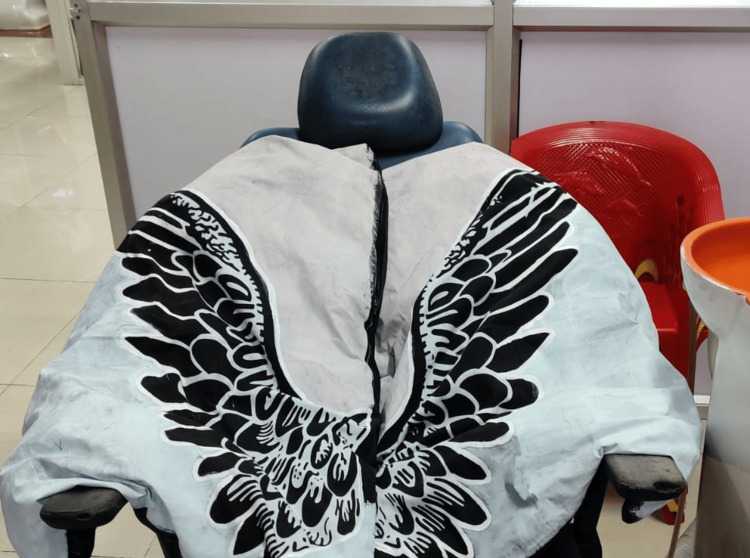
Weighted lap pad in the shape of angel wings The weighted lap pad was made using cotton breathable fabric, which was non-irritant to the patient, and filled with cotton pieces.

**Figure 3:**
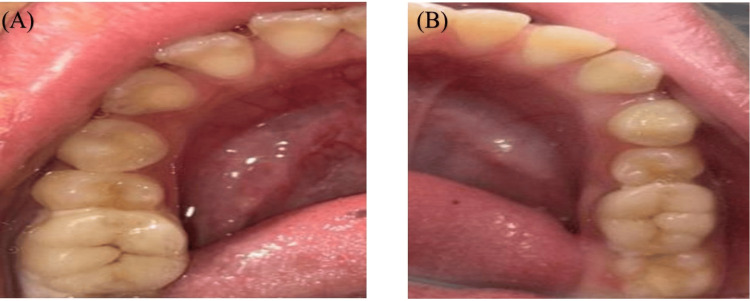
Postoperative intraoral photographs showing zirconia crowns on the mandibular first permanent molars (A) Zirconia crown luted in the left mandibular first permanent molar. (B) Zirconia crown luted in the right mandibular first permanent molar.

Dental prophylaxis was performed using a gentle, step-by-step approach to minimize discomfort and anxiety for the patient. The dental operatory was modified to reduce sensory stimuli by dimming the overhead lights and playing calming music. A weighted lap pad (in the shape of angel wings) was offered to help her feel more comfortable and grounded during the appointment. Soft foam sensory toys were provided. Figure 2 illustrates the weighted lap pad shaped like angel wings. It was made from breathable cotton fabric, which was non-irritating to the patient, and filled with cotton pieces. Positive reinforcement techniques, such as praise and token rewards, were used to encourage her cooperation during the procedure. Desensitization strategies, including gradual exposure to the dental chair, dental instruments, and tactile stimuli, were employed to help her become more comfortable with the dental environment. Finally, the mouth mirror was used to examine the oral cavity. Cheek retractors were used to help her keep her mouth open after confirming her comfortability. The trial and fit for zirconia crowns were done. Occlusion was checked using mouth mirrors. Luting of the zirconia crown was done using type 1 glass ionomer cement in the right and left first mandibular molars. Postoperative instructions were given to the patient. Figure 3 presents a postoperative clinical photograph of the oral cavity.

**Figure 4:**
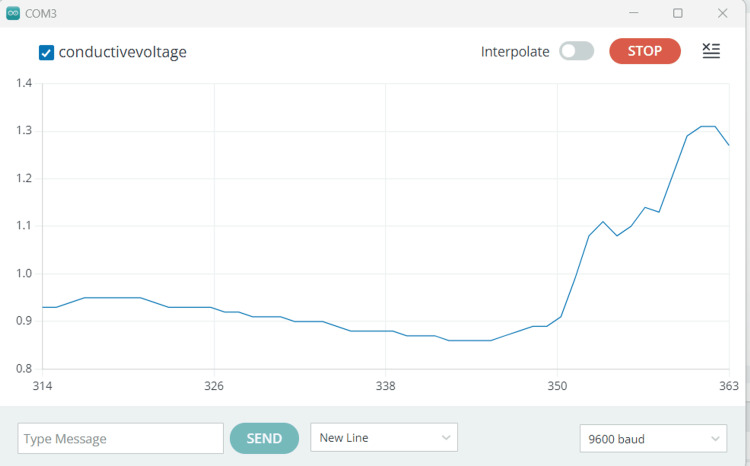
Graph showing the EDA response of the first patient The graph depicts the changes in EDA from time points 314 to 363. The y-axis represents the parameter value ranging from 0.8 to 1.4, while the x-axis denotes the time points. EDA: electrodermal activity

**Figure 5:**
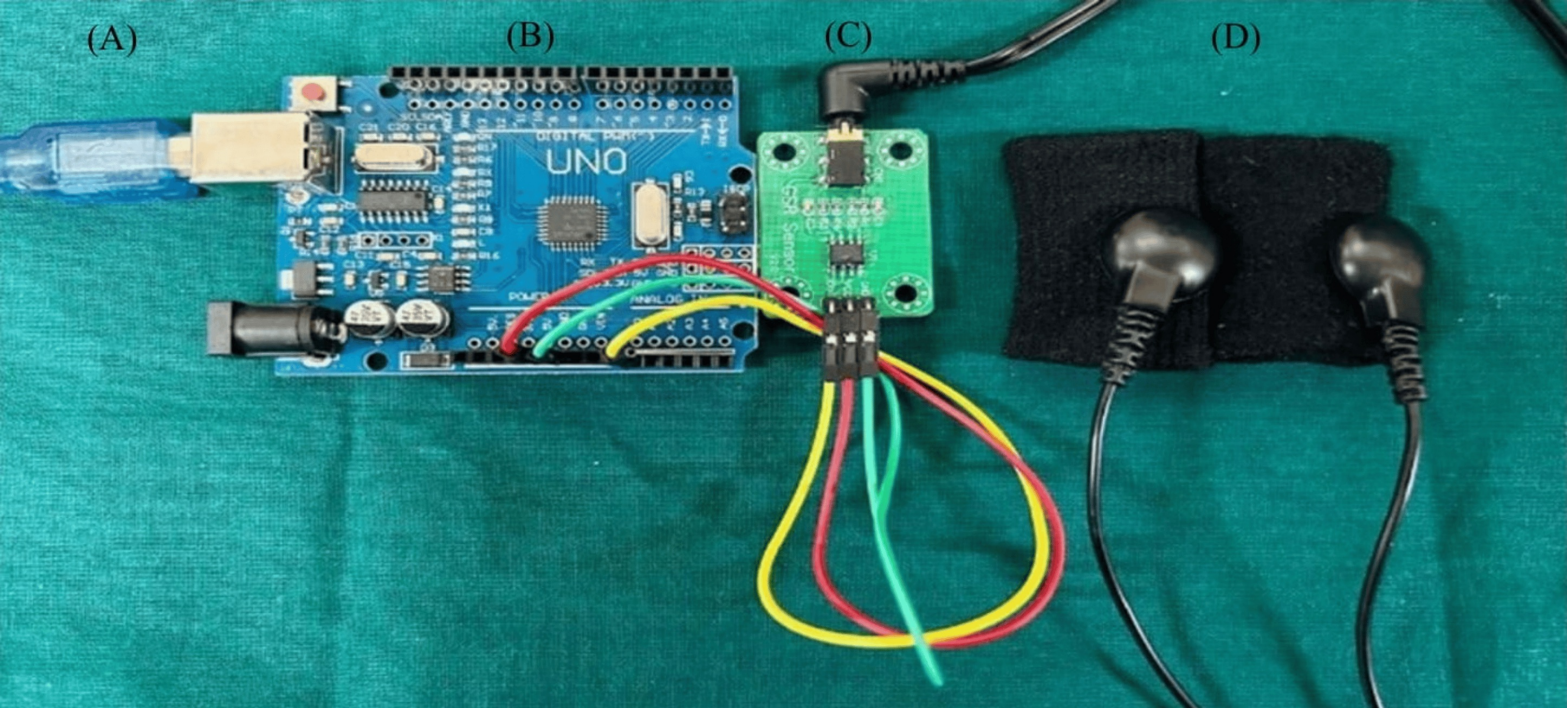
Galvanic skin response device for recording EDA This device includes the following components: (A) a USB cable, (B) a microcontroller board (Arduino Uno), (C) a GSR sensor module, and (D) electrodes. USB: universal serial bus, GSR: galvanic skin response, EDA: electrodermal activity

EDA was continuously recorded using pre-gelled electrodes on the child’s non-dominant hand’s index and middle fingertips for three minutes before and during the dental procedure. Phasic variations in EDA levels were noted throughout the treatment when she was exposed to stressful stimuli, in this case, bright lights and loud sounds. Figure 4 depicts a graph showing the EDA response of the first patient. The device for recording EDA, known as the galvanic skin response device, is depicted in Figure 5.

Case 2

Patient Description

An eight-year-old boy with moderate early childhood autism presented to the Department of Pediatric and Preventive Dentistry with the chief complaints of inadequate oral hygiene habits and noticeable plaque buildup on his anterior teeth. The child’s mother, in her capacity as his guardian, was also present during the consultation. The boy had good speech comprehension; however, verbal communication was not practical as he did not speak and limited his communication to facial expressions.

Remarkably, he displayed very little, if any, resistance to the lights, sounds, and odors in the SADE, perhaps because the music coming from the speakers brought him some happiness. The parent received a thorough explanation of the oral examination analysis.

Treatment Plan

His parents received social narratives and visual aids outlining the dentist visit procedure before the appointment. Because the child was nonvocal and pleasantly responded to the music, behavioral techniques such as the "Tell-Show-Do" technique were implemented using dentist songs in his regional language [7]. The dental operatory was modified to reduce sensory stimuli, which included changing light intensity around the dental chair, playing music best suited to the patient's liking, and assisting in his cooperation in the dental chair. A weighted lap pad (in the shape of angel wings) was offered to him to help him feel more comfortable during the appointment, but the patient rejected it because of his over-responsivity to touch stimuli.

The use of positive reinforcement tactics, including playing his choice of music and expressing appreciation for his cooperation, was implemented to foster his participation throughout the process. To help him acclimate to the dental environment, desensitization techniques were used, including gradual exposure to dental equipment and tactile stimuli. A firm tone of voice was maintained throughout the treatment. Each technique was selected based on its appropriateness for addressing the patient’s needs and enhancing their overall experience.

**Figure 6:**
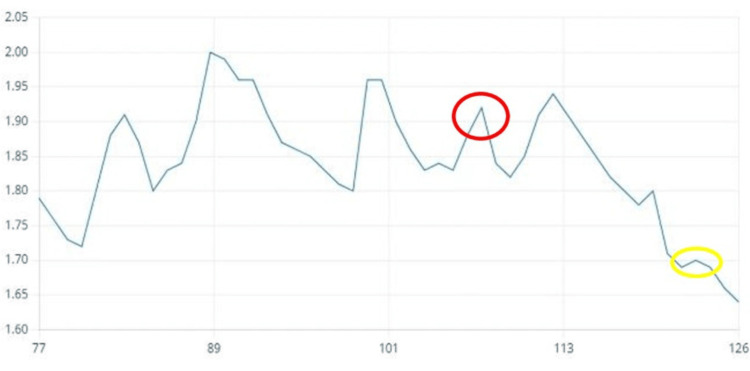
Graph showing the EDA response of the second patient The graph depicts the changes in EDA from time points 77 to 126. The y-axis represents the parameter value ranging from 1.65 to 2.05, while the x-axis denotes the time points. Phasic change: red circle, tonic change: yellow circle, EDA: electrodermal activity

EDA was continuously recorded during the dental procedure and for three minutes before the oral prophylaxis. Pre-gelled electrodes were applied to the index and middle fingertips of the child’s non-dominant hand. Figure 6 shows phasic changes in the presence of stressful stimuli, such as the weighted lap pad and strange surroundings, represented in red, and tonic changes when calming stimuli, such as music, preferably the child’s own selection, are present, represented in yellow. The graph depicts the changes in EDA from time points 77 to 126. The y-axis represents the parameter value ranging from 1.65 to 2.05, while the x-axis denotes the time points.

**Figure 7:**
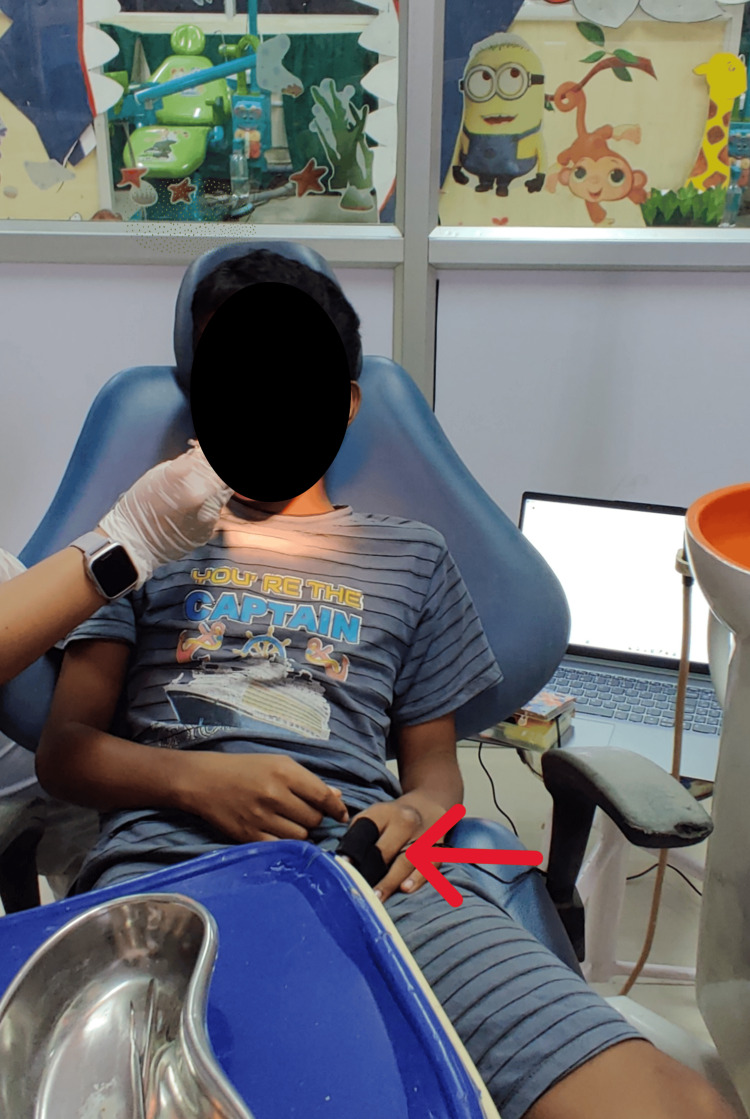
EDA recording during a dental procedure EDA was continuously recorded using pre-gelled electrodes placed on the index and middle fingertips of the child's non-dominant hand (indicated by the red arrow) for three minutes before and during the dental procedure. EDA: electrodermal activity

**Figure 8:**
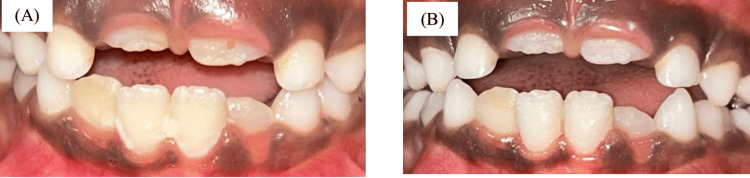
Clinical photographs (A) Preoperative clinical photograph showing the oral cavity of an eight-year-old boy with ASD requiring scaling of his anterior teeth. (B) Postoperative clinical photograph showing the oral cavity of an eight-year-old boy with ASD after hand scaling. ASD: autism spectrum disorder

Figure 7 shows an in-procedure photograph of the patient with EDA being recorded during the dental procedure. A calm, methodical approach was taken during dental prophylaxis to reduce the patient’s level of discomfort and anxiety. Because ultrasonic scalers might result in sensory overload, manual scaling was performed instead. Figure 8 shows preoperative and postoperative clinical photographs of the patient's oral cavity.

Case 3

Patient Description

A six-year-old child with moderate ASD presented to the Department of Pediatric and Preventive Dentistry with complaints of stains in the mandibular lingual region and overall unmanageable oral hygiene. The patient was accompanied by his mother and grandfather at the time of the appointment. The mother reported that her child exhibited selective eating habits, often referred to as "picky eating," where he consumed a limited variety of foods and refused to try new ones. She also noted his aversion to bright lights and sudden touches, as well as his affinity for music and calm sounds.

Treatment Plan

Prior to the appointment, his mother was provided with sensory toys and social stories explaining the stimuli occurring during dental treatment. Behavioral techniques such as the "Tell-Show-Do" technique were applied, and dentist-themed songs in the local language were utilized.

The dental operatory was modified to reduce sensory stimuli; there were no bright lights, and music was played using headphones. Weighted lap pads were not offered to him because he was hyper-responsive to touch stimuli.

The use of positive reinforcement tactics, including the music of his choice played in the headphones and an oral massager, was used to calm hyperstimulation, as the patient exhibited oral fixation and found relief from anxiety and stress through the use of an oral massager. According to the patient's mother, the massager was a preferred method of self-soothing. The "Tell-Show-Do" method was used to explain and demonstrate procedures. Desensitization was applied to gradually reduce anxiety, and voice control, which was steady, friendly, and reassuring, was utilized to manage the patient's comfort and behavior. Each technique was selected based on its appropriateness for addressing the patient’s needs and enhancing their overall experience.

**Figure 9:**
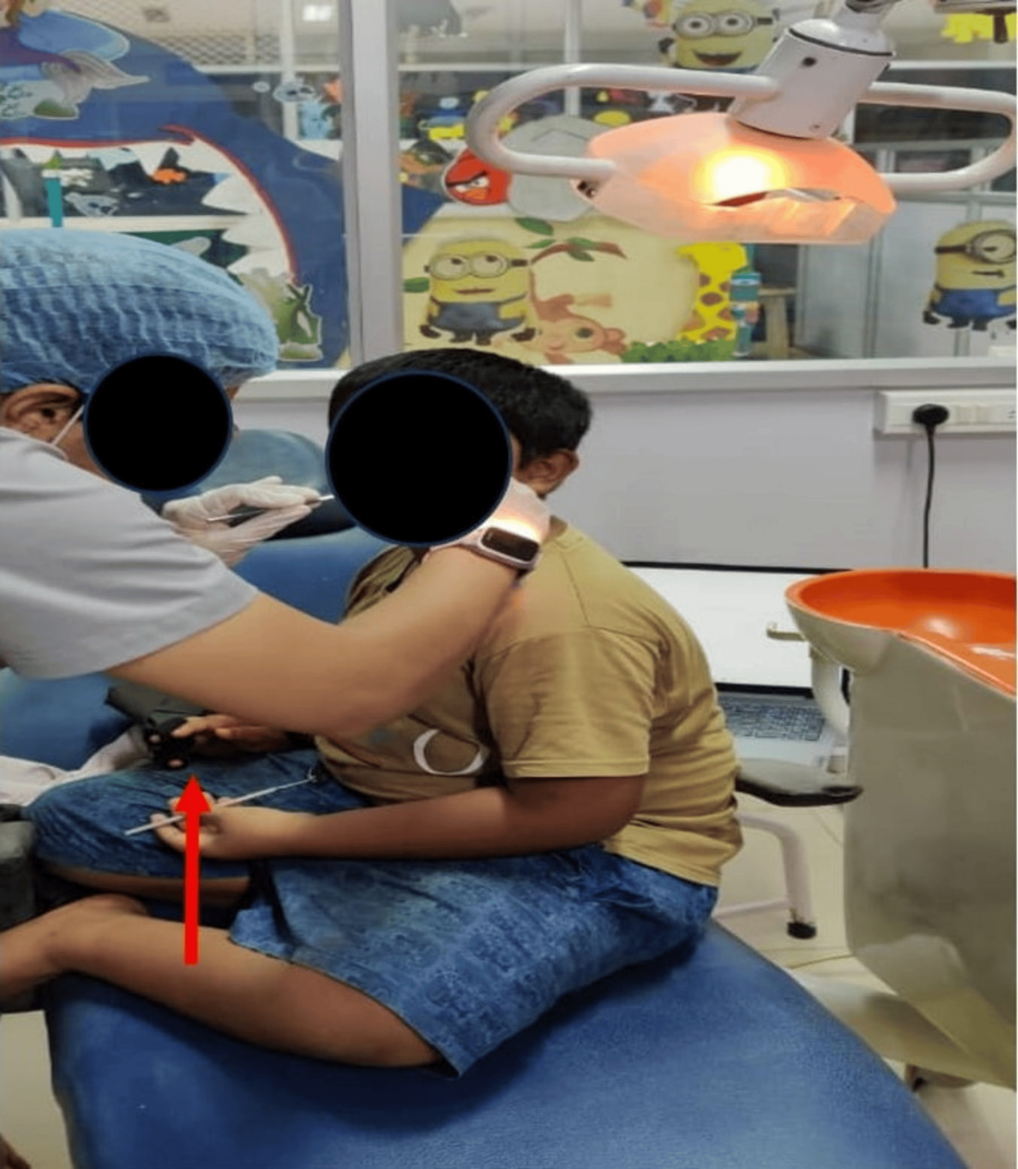
EDA recording during a dental procedure EDA was continuously recorded using pre-gelled electrodes placed on the index and middle fingertips of the child's non-dominant hand (indicated by the red arrow) for three minutes before and during the dental procedure. EDA: electrodermal activity

**Figure 10:**
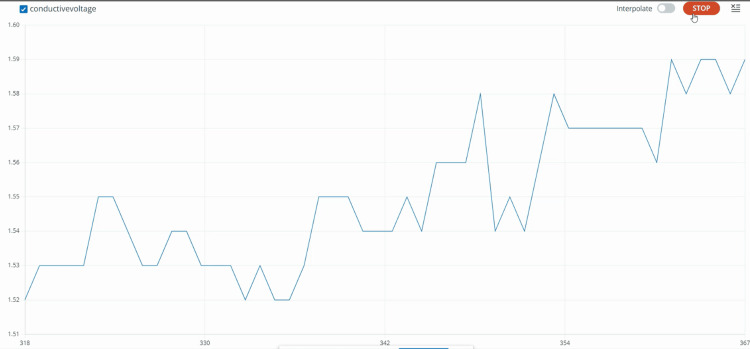
Graph showing the EDA response of the third patient The graph depicts the changes in EDA from time points 318 to 367. The y-axis represents the parameter value ranging from 1.51 to 1.60, while the x-axis denotes the time points. EDA: electrodermal activity

**Figure 11:**
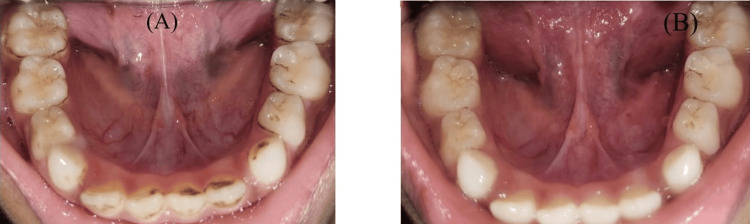
Clinical photographs (A) Preoperative clinical photograph showing the oral cavity of an eight-year-old boy with ASD requiring scaling of his mandibular teeth. (B) Postoperative clinical photograph showing the oral cavity of an eight-year-old boy with ASD after hand scaling. ASD: autism spectrum disorder

EDA was continuously recorded during the dental procedure and for three minutes before the oral prophylaxis. Figure 9 shows an in-procedure photograph of the patient with EDA being recorded during the dental procedure. Pre-gelled electrodes were applied to the index and middle fingertips of the child’s non-dominant hand. During the treatment, noticeable phasic changes in EDA levels occurred in response to stressful stimuli, such as confined spaces and sudden exposure to light and touch. Conversely, tonic changes were observed during calming stimuli, including music played through headphones and the use of a massager. Figure 10 displays the EDA for the patient. It depicts the changes in EDA from time points 318 to 367. The y-axis represents the EDA value, ranging from 1.51 to 1.60, with increments of 0.01, while the x-axis denotes the time points. A calm, methodical approach was adopted during the dental prophylaxis to minimize the patient's discomfort and anxiety. To avoid sensory overload from ultrasonic scalers, manual scaling was performed instead of using scaler tips. Figure 11 shows preoperative and postoperative clinical photographs of his oral cavity.

## Discussion

ASD is a neurodevelopmental condition that affects how an individual perceives and engages with others, leading to difficulties in social interaction and communication. According to the Centers for Disease Control and Prevention, ASD is a neurodevelopmental disability attributed to brain differences [8]. The diagnosis of ASD is often made in early childhood between the ages of 18 and 24 months, and its frequency has been rising over time [8]. Between 2012 and 2021, the percentage of new and old cases of ASD worldwide grew from 0.62% to 1.0% [8].

Dental prophylaxis for children with autism requires a patient-centered approach that considers the individual’s sensitivity to sensory stimuli, communication abilities, and behavioral challenges [9]. Children with ASD can receive effective dental care that minimizes stress and promotes oral health by establishing a supportive environment and putting customized strategies into practice [9]. According to Kuhaneck et al., patients with ASD may have better dental visits and more access to dental care if their dentists are aware of sensory processing disorders and are knowledgeable about techniques to improve the sensory experience [9].

Reports by other studies of individuals with impairments have demonstrated the significance of delivering the information beforehand. Additionally, the educators at the Special Education Center state that this knowledge has to be given visually, using a picture album or other comparable resources, due to the peculiarities of children with autism [10]. In our case series, we provided parents with sensory toys and social stories before the appointment to explain the stimuli that would be encountered during the dental treatment.

Methods such as desensitization and video modeling, employed in our study, have proven to be effective tools for behavior management. To achieve the desired results in practical dentistry settings, the authors agree that it is crucial to apply a combination of established behavior management techniques. The perspectives of authors like Lewis align with the observation that there is no "one size fits all" approach to dental and oral care for children with ASD [10].

Both physically and behaviorally, children with ASD differ in how they receive sensory information [10-12]. Prince et al. showed EDA to be a useful indicator of individual diversity in ASD and early development [13]. People on the autism spectrum may experience dysregulated or heightened sympathetic reactions, particularly during social interactions, making these activities unpleasant [13]. EDA includes both background tonic (SCL) and rapid phasic components (SCRs) that result from sympathetic neuronal activity. The tonic component, represented by SCL, relates to the slower-acting components and background characteristics of the signal, such as the overall level and slow changes over time. Changes in SCL reflect general autonomic arousal. The phasic component, represented by SCR, refers to the faster-changing elements of the signal. Recent evidence suggests that both SCL and SCR are important and may rely on different neural mechanisms [5]. EDA is arguably the most useful index of changes in sympathetic arousal related to emotional and cognitive states, as it is the only autonomic psychophysiological variable not contaminated by parasympathetic activity. EDA has been closely linked to autonomic, emotional, and cognitive processing and is widely used as a sensitive index of these processes and sympathetic activity. Variations in SCL and SCR are associated with affect, novelty, sensory input, anxiety, attention, and cognitive demand [13].

In our case series, all three patients exhibited phasic variations in EDA levels throughout the treatment when exposed to stressful stimuli. Tonic changes were observed when calming stimuli were present. The response to stressful and calming stimuli varied among the three patients. Case 1 showed phasic variations in response to bright lights and unfamiliar settings, while Case 2 exhibited similar responses to a weighted lap pad. Case 3 reacted to confined spaces and sudden exposure to light and touch. Tonic phases were observed as firm pressure for Case 1, music for Case 2, and both a massager and music for Case 3.

To enhance patient cooperation, we addressed these stressors by using dim lighting, avoiding loud music, and keeping doors closed to minimize sudden noises. Conversely, when calming stimuli, such as firm pressure, were applied, tonic changes in EDA were noted. Therefore, we maintained consistent firm pressure and avoided sudden touches to improve patient comfort according to each patient's specific stressors.

This case series is a medium for exploring and understanding the relationship between sympathetic arousal and ASD and its association with dental treatment in a sensory-adapted dental environment. With collaborative efforts, occupational therapists and dentists can design changes to the regular dental environment or modify dental protocols to lessen some of the obstacles faced by individuals with ASD [6].

## Conclusions

Dental care for children with autism requires a patient-centered approach that considers their sensitivity to sensory stimuli, communication abilities, and behavioral challenges. EDA, which measures changes in sympathetic arousal, has been useful in understanding individual differences in ASD. Our case series showed that patients exhibited phasic variations in EDA levels in response to stressful stimuli and tonic changes with calming stimuli. Strategies to enhance patient cooperation included using dim lighting, avoiding loud noises, and applying firm pressure, along with providing sensory toys and social stories before appointments and employing desensitization and video modeling techniques. This case series highlights the importance of adapting dental environments and protocols to meet the needs of individuals with ASD and facilitate their cooperation during dental treatment.
